# The dopamine hypothesis of bipolar affective disorder: the state of the art and implications for treatment

**DOI:** 10.1038/mp.2017.16

**Published:** 2017-03-14

**Authors:** A H Ashok, T R Marques, S Jauhar, M M Nour, G M Goodwin, A H Young, O D Howes

**Affiliations:** 1 Psychiatric Imaging Group, MRC London Institute of Medical Sciences, Hammersmith Hospital, Imperial College London, London, UK; 2 Psychiatric Imaging Group, Institute of Clinical Sciences (ICS), Faculty of Medicine, Imperial College London, London, UK; 3 Department of Psychosis Studies, Institute of Psychiatry, Psychology and Neuroscience, Kings College London, London, UK; 4 South London and Maudsley NHS Foundation Trust, Camberwell, London, UK; 5 Department of Psychiatry, University of Oxford and Oxford Health NHS Trust, Warneford Hospital, Oxford, UK; 6 Department of Psychological Medicine, Institute of Psychiatry, Psychology and Neuroscience, King's College London, London, UK

## Abstract

Bipolar affective disorder is a common neuropsychiatric disorder. Although its neurobiological underpinnings are incompletely understood, the dopamine hypothesis has been a key theory of the pathophysiology of both manic and depressive phases of the illness for over four decades. The increased use of antidopaminergics in the treatment of this disorder and new *in vivo* neuroimaging and post-mortem studies makes it timely to review this theory. To do this, we conducted a systematic search for post-mortem, pharmacological, functional magnetic resonance and molecular imaging studies of dopamine function in bipolar disorder. Converging findings from pharmacological and imaging studies support the hypothesis that a state of hyperdopaminergia, specifically elevations in D2/3 receptor availability and a hyperactive reward processing network, underlies mania. In bipolar depression imaging studies show increased dopamine transporter levels, but changes in other aspects of dopaminergic function are inconsistent. Puzzlingly, pharmacological evidence shows that both dopamine agonists and antidopaminergics can improve bipolar depressive symptoms and perhaps actions at other receptors may reconcile these findings. Tentatively, this evidence suggests a model where an elevation in striatal D2/3 receptor availability would lead to increased dopaminergic neurotransmission and mania, whilst increased striatal dopamine transporter (DAT) levels would lead to reduced dopaminergic function and depression. Thus, it can be speculated that a failure of dopamine receptor and transporter homoeostasis might underlie the pathophysiology of this disorder. The limitations of this model include its reliance on pharmacological evidence, as these studies could potentially affect other monoamines, and the scarcity of imaging evidence on dopaminergic function. This model, if confirmed, has implications for developing new treatment strategies such as reducing the dopamine synthesis and/or release in mania and DAT blockade in bipolar depression.

## Introduction

Bipolar disorder (BD) is a severe mental illness characterised by recurrent episodes of mania, depression or mixed states.^1, 2^ The lifetime prevalence of the full spectrum of bipolar disorder is estimated to be 2–4% in the general population and it is the sixth leading cause of disability worldwide.^3, 4, 5, 6^ The costs due to BD are immense, with annual direct healthcare costs in the USA of about $30 billion and indirect costs of >$120 billion.^7, 8^

Lithium has been the mainstay of maintenance treatment for BD for decades, together with valproate, an option that has emerged more recently. However their modes of action have not been well established. By contrast antidopaminergic drugs do have well-understood class action at D2/3 receptors and have long been used to treat acute manic episodes. However, as will be explained below, newer drugs (particularly olanzapine and quetiapine) have been shown to have antimanic and antidepressant actions in bipolar disorder together with long-term benefit in preventing relapse to either pole of the illness.^9, 10, 11, 12, 13, 14, 15^ Although the newer drugs were introduced for the treatment of schizophrenia, they have arguably represented a bigger advance for the management of bipolar disorder.

Despite these advances in treatment, many patients continue to experience high levels of disability.^11^ Furthermore drugs may be associated with significant side-effect burden and the risk of toxicity and/or teratogenicity in some instances.^16, 17, 18^ Hence better understanding of pathophysiology and drug action appears necessary to improve the use of current treatments and develop better alternatives.

The dopamine hypothesis of BD dates back at least to the 1970s.^19, 20, 21^ Early incarnations focused on mania, and the parallels between the behavioural consequences of amphetamine ingestion and the antimanic actions of antidopaminergic drugs. If hyperdopaminergia underlies the development of manic symptoms, then hypodopaminergia might underlie the depressive phase of the illness. Thus, opposite changes in dopaminergic function were hypothesised to underlie the opposing affective poles of the disorder.^19, 20, 21^ This theory did not explain how hyper- or hypodopaminergia would arise and subsequent versions proposed an additional component to the hypothesis, where an intrinsic dysregulation in the homoeostatic regulation of dopaminergic function leads to cyclical changes in dopaminergic neurotransmission,^22, 23^ which would further distinguish the dopamine hypothesis of bipolar from theories of schizophrenia.^24^ Thus, faulty homoeostatic mechanisms responding to hyperdopaminergia in the manic phase of the illness are proposed to result in an excessive reduction in dopaminergic function, rapidly leading to a hypodopaminergic state and depression. In turn a faulty regulatory response to hypodopaminergia leads, to a switch back to mania.^22, 23^ Implicit in this model is that a normalisation of dopaminergic function leads to remission and euthymia. Clearly, some kind of dysregulation must be required to account for the onset of episodes, but BD is also characterised by inter-episode mood instability.^25^ Any satisfactory theory must account for the randomness of much bipolar experience as well as the cyclicity. As current antimanic antipsychotics are all dopamine D2 receptor blockers, their use for mania has long supported the involvement of dopamine in mania, but the increasing use of some antidopaminergic drugs to treat bipolar depression and as maintenance drugs makes it timely to review the dopamine hypothesis of BD. Furthermore, a number of new lines of evidence relevant to the role of dopamine, in particular from molecular and functional neuroimaging, have developed in recent years. Thus, we synthesize evidence from pharmacological, *in vivo* neuroimaging and post-mortem studies addressing the role of the dopamine system in BD and then consider the treatment and drug development implications.

## Animal models and their implications for understanding the role of dopamine in bipolar disorder

Initial animal models of mania were based on amphetamine-induced hyperlocomotion, and it was shown that lithium reverses this behaviour.^26, 27^ Following this, a dopamine transporter (DAT) knockout rodent model was developed, and dopamine transporter blockers other than amphetamine (such as GBR12909) have been used. Both these approaches have been shown to induce manic-like behaviour, such as hyperlocomotion and increased exploration.^28, 29^ Subsequently, dopamine receptor stimulation using quinipirole (a dopamine agonist) was shown to induce manic-like behaviour.^30^ Interestingly, mood stabilisers such as valproate and carbamazepine reversed these effects.^30^ Recently, Sidor and colleagues demonstrated the impact of alterations in the regulation of circardian rhythm on dopaminergic activity and behaviour.^31^ In this study, mice with a mutation in a circadian clock gene displayed a manic-like phenotype as measured by hyperlocomotion in the daytime. Moreover, hyperlocomotion was linked to an elevated daytime spike in ventral tegmental area (VTA) dopaminergic activity, as well as increased dopamine synthesis and tyrosine hydroxylase activity.^31^ This was further validated using sustained optogenetic stimulation of the VTA, which also induced hyperlocomotion behaviour.^31^ Taken together this evidence suggests that hyperdopaminergia, induced either by increased dopamine release, dopamine transporter blockade or dopamine receptor stimulation, results in a mania-like phenotype in rodents. In contrast, lesions in dopaminergic areas (such as the VTA and substantia nigra) induce depressive behaviour, such as learned helplessness.^32^ Moreover, optogenetic stimulation of VTA dopaminergic neurons reverses depressive behaviour (as assessed using the forced swim test in chronically stressed animals).^33^ These models thus provide evidence that hypodopaminergia may induce depressive-like behaviours and hyperdopaminergia may induce manic-like behaviour. Further research is needed to determine the key components of the dopaminergic circuit underlying this, particularly in relation to depressive behaviour.

## Post-mortem studies on dopamine gene expression in bipolar disorder

Table 1 shows the post-mortem studies addressing dopaminergic gene expression in BD. Overall the most consistent findings come from studies on the D2 receptor expression, with two studies suggesting that the D2 receptor is upregulated in BD in the dorsolateral prefrontal cortex.^34, 35^ However, to date only four studies,^28, 30,34,36^ have been conducted, and only two focus on the same region (dorsolateral prefrontal cortex). Thus, this finding still requires replication in other brain regions to determine if there is regional specificity. The literature on other dopaminergic receptors is more limited, with only two studies on the D1 receptor^34, 37^ and one study on the D5 receptor.^38^ Another issue for the post-mortem studies is that they are affected by several confounding factors such as medication status, post-mortem interval, substance abuse and cause of death. Antidopaminergic treatment has been shown to increase D2/3 receptor levels in animals,^39^ and there is evidence this may also occur in schizophrenia.^40^ This suggests that prior treatment could also have affected the D2/3 receptor findings in BD. Finally, data on phase-specific changes are difficult to characterise as clinical status at the time of death remains unknown. Despite these limitations, post-mortem data supports the notion that an abnormality within the dopaminergic pathways, in particular involving D2/3 receptors, might play a role in the pathogenesis of BD.

## *In vivo* imaging of dopamine

### Dopamine in mania

We have summarised the *in vivo* imaging studies of mania in Table 2. Interestingly, patients with psychotic mania showed an elevated density of D2/3 receptors as measured by N-[^11^C]-methylspiperone, when compared with healthy controls (HC) and non-psychotic mania patients,^41, 42^ although, as this tracer has significant affinity for 5HT2 receptors as well,^43^ this finding requires replication with more selective tracers. Moreover, no significant difference in the striatal D2/3 density was noted in non-psychotic mania patients compared to HC.^44^ These studies also explored the relationship between manic symptoms (as assessed using Young's Mania Rating Scale Score) and dopamine synthesis capacity and D2/3 density, finding no significant correlations between these variables in patients with mania.^41, 44, 45^ However, in one of these studies D2/3 density was directly correlated with psychosis scores on the present state examination.^41^ Taken together, these data suggest that psychotic symptoms in mania may be associated with dopaminergic abnormalities, although the same cannot be inferred in non-psychotic mania patients.

### Dopamine in euthymic BD

Two studies have assessed dopamine transporters in the euthymic state and are conflicting. Chang *et al* reported an upregulation of the dopamine transporter in drug naive euthymic bipolar patients, while Anand *et al.* observed a downregulation in a group consisting of both euthymic bipolar and bipolar depression patients.^46, 47^ Additionally, one study has assessed the vesicular monoamine transporter protein (VMAT-2), located in presynaptic neurons and involved in the storage of dopamine. There was an increase in VMAT-2 in the thalamus and dorsal brainstem, but no significant alteration in striatum, in currently euthymic patients with a history of psychotic mania compared with HC.^48^ A positron emission tomography (PET) study using [^11^C]-SCH23390 found decreased D1 density in the frontal cortex but not in the striatum of bipolar patients compared to HC, suggesting a regional difference in D1 receptor distribution (Table 3).^49^

So far, only one PET study has assessed dopamine release in BD. This study used [^123^I]-IBZM to measure dopamine receptor density and release after amphetamine challenge in patients in their euthymic phase of illness, and found no significant differences between patients and HC.^50^

### Dopamine in bipolar depression

There is a paucity of literature on dopamine release and post-synaptic dopamine receptor density. Findings on dopamine transporter density remains controversial, with one study showing an increase in bipolar depressive patients when compared with HC, whilst the others, which recruited both euthymic and depressed patients, noted a reduction (Table 3).^21, 26^

## Dopamine, reward processing and bipolar disorder: functional magnetic resonance imaging studies

Dopaminergic projections from the VTA to the ventral striatum (VS) (including nucleus accumbens) and prefrontal cortex have an important role in reward processing^51, 52^ and have also been implicated in the processing of other behaviourally salient stimuli.^53, 54^ The word 'reward' implies three related functional elements: a positive reinforcer for learning, movement towards a desired object and subjective/emotional choices. The brain processes underlying reinforcement and movement can be quantitatively assessed using specific behavioural tasks and single cell or optogenetic recording in animals.^55^ Tasks that inform emotion *per se* are increasingly being performed in man but necessarily with less direct measures of neuronal activity.

Investigations in animals have shown that individual neurons signal reward-related information in the midbrain (substantia nigra and VTA), striatum, orbitofrontal cortex, amygdala and associated structures. Most dopamine neurons in the substantia nigra and VTA show brief, phasic responses that reflect the difference in value between received reward and predicted reward. The precise relationship between cell firing and function is still debated, but it would be misleading to think of dopamine neurotransmission simply in terms of a pleasure signal.^56^ Changes in BOLD signal occur in the human nucleus accumbens, striatum and frontal cortex over a time course of seconds in response to anticipated reward.^57^ Consistent with the animal literature, event-related functional magnetic resonance imaging (fMRI) reward paradigms have demonstrated robust activation of the ventral striatum in relation to both the anticipation and receipt of reward,^58, 59, 60, 61^ presumably related to dopaminergic neurotransmission.^57, 62, 63, 64, 65, 66, 67, 68, 69^

In BD several fMRI studies employing reward tasks support the existence of abnormal reward-related neural activity in the VS and frontal cortex (task paradigms and results for VS summarised in Supplementary Table 2 and Table 4, respectively).^61, 70, 71, 72, 73, 74, 75, 76, 77, 78, 79, 80, 81, 82^ This provides another indirect line of evidence for a dopaminergic abnormality in this disorder. We discuss the evidence in relation to specific mood polarities below.

### Bipolar mania

During cued reward anticipation Abler *et al.* reported that medicated manic patients had reduced activity to high- vs no-anticipated reward in the VTA,^70^ owing to increased neural activity for no-reward-predicting cues in BD patients. Three studies using monetary reward tasks found no difference in VS activation to cued reward anticipation between patients and controls.^70, 71, 80^ Two of these studies did, however, find elevated frontal cortex activity during reward anticipation in manic patients.^71, 80^ During reward feedback Abler *et al.* reported reduced activity in response to receipt of probabilistic rewards in the VS in BD compared with HC.^70^ This finding has not been replicated in more recent studies in patients with current manic symptoms^71^ or a recent manic episode.^80^

Together, these studies suggest hyperactive neuronal activity in putatively dopaminergic circuits of the reward system in currently manic patients, particularly during cue-induced reward anticipation. The precise nature of this abnormality, however, remains uncertain.

### Euthymic bipolar

Euthymic patients are of particular interest because they are at risk of mania. In these patients abnormalities in dopaminergic function would then indicate how vulnerability might be mediated. In medicated euthymic bipolar I patients, VS and prefrontal cortex activity to cued reward anticipation has been variously reported as increased,^76, 77^ and no different^72, 74^ compared with controls. During reward feedback VS activity in euthymic bipolar I patients has been reported as increased,^72, 74, 76^ no different^75, 77^ and decreased (in euthymic and mildly depressed patients),^81^ compared with controls. Reward feedback related activation in the frontal cortex has also variously been reported as elevated,^75, 76^ decreased (in euthymic and mildly depressed patients)^81^ or no different^74, 77^ in bipolar I patients compared with controls. In euthymic bipolar II patients Caseras *et al.* reported elevated VS and prefrontal cortex activity for cued reward anticipation compared with controls.^72^ This finding, however, was not replicated in a more recent study of medication-naive bipolar II patients, which instead reported reduced anticipation-related activity in the right dorsal striatum in patients.^82^ Both studies reported no difference in VS activity during reward feedback in patients vs controls. Increased responses in the anticipatory phase of the monetary incentive delay task have been described in euthymic unipolar patients.^83^ Careful comparison of unipolar with bipolar cases will be necessary to distinguish network dysfunction associated with (hypo)mania from that with depression. All such studies need to be adequately powered and preferably address pre-specified hypotheses, not always a standard observed in imaging studies.

In summary, the precise nature of the abnormality of reward-related activity in euthymic bipolar patients remains unclear. However, abnormalities of reward processing are probably not confined to the manic phase of the illness. Important differences in task structure and the *post hoc* choice of analysis contrasts between these studies may account for the inconsistencies and non-replication. Further studies are required to explore the suggested differences between euthymic bipolar I, bipolar II and unipolar disorder.

### Bipolar depression

Three studies have compared monetary reward processing in medicated depressed bipolar I patients and HC. Only one study analysed activity during cued reward anticipation; it reported no difference in anticipation-related activity between patients and HC in VS or prefrontal cortex, although there was blunting in the anterior cingulate cortex in depressed patients (bipolar and unipolar). Neural activity related to outcome anticipation *per se* was increased in left ventrolateral prefrontal cortex in patients with bipolar-1 disorder, compared with healthy controls or patients with unipolar depressive disorder; it may represent a more generalised ‘arousal' response in the bipolar depressed group.^73^ Ventral striatal and prefrontal activation during reward feedback in bipolar depressive patients has been reported both as similar to controls^73, 79^ and decreased.^78^

Recent fMRI studies and meta-analyses have demonstrated blunted activation of the reward network in the ventral striatum and frontal cortex in schizophrenia,^84, 85, 86^ as well as major depressive disorder and alcohol addiction.^84^ In contrast, the fMRI findings in bipolar disorder summarised above do not provide consistent evidence for blunting. It may be important to consider only studies in the manic state, because depressed or even euthymic (often dysthymic) bipolar patients may well show blunting.^82^ In mania there was increased frontal activation to reward anticipation in two out of three studies. If this finding is confirmed in further studies, including direct comparisons with patients with schizophrenia, it could point to a key difference in the reward network between mania and schizophrenia. To date a small number of studies have compared reward processing in bipolar disorder with schizophrenia^70^ or major depressive disorder.^73, 78^ Of these, one study reported blunted VS activation in depressed patients diagnosed with bipolar disorder compared with major depressive disorder, at reward feedback.^78^ Only one study compared reward processing in schizophrenia and manic patients with controls.^70^ Patients with schizophrenia and healthy controls showed an activation in the VTA on expectation of monetary rewards and nucleus accumbens activation during receipt vs omission of rewards. Manic patients, however, showed reduced differential activation in the nucleus accumbens on receipt vs omission of rewards compared to the healthy control subjects. Taken together with the findings in the frontal cortex discussed above, this small study does suggest that in mania there is a deficit in prediction error processing not seen in schizophrenia. It may further imply bipolar disorder specific changes in dopaminergic function, although how much of the fMRI response is driven by disrupted dopaminergic function remains to be determined. Further studies investigating differences in reward processing between bipolar disorder and other psychotic and affective disorders, and across illness phases, will shed light on the reward processing abnormalities specific to bipolar disorder.

## Pharmacological evidence

### Dopaminergic manipulations and the induction of mania

The role of dopaminergic abnormalities in BD has been extensively investigated using a variety of pharmacological approaches (Table 5). Firstly, studies have shown that psychostimulants, particularly amphetamine, cause mania-like symptoms in healthy volunteers.^87, 88, 89, 90^ Further, there are several case series of manic and hypomanic episodes in Parkinson's disease patients treated with Levodopa.^91, 92, 93^ Bromocriptine, another dopamine agonist, has also been shown to induce manic symptoms.^94, 95^ Similarly, an increased risk of hypomania/mania was observed in bipolar patients who received stimulant (methylphenidate, amphetamine or modafinil) augmentation for bipolar depression.^96^ Secondly, pharmacological strategies to deplete tyrosine, a dopamine precursor, are known to reduce manic symptoms, although it remains to be established if this is specifically due to reducing dopamine levels.^97, 98^ Alpha-methyl-p-tyrosine, which induces dopamine depletion by inhibition of tyrosine hydroxylase, also attenuates mania-like symptom in bipolar patients.^99, 100^

### Dopaminergic manipulations and the induction of bipolar depression

Reserpine and tetrabenazine depletes synaptic dopamine by irreversibly inhibiting vesicular uptake of monoamines and has long been known to induce depression.^101, 102^ In addition, a high prevalence of depression is seen in patients with Parkinson's disease and this has been linked to loss of striatal dopaminergic innervation.^103, 104^ Finally, depletion of tyrosine has been shown to precipitate depressive symptoms in remitted patients with a history of major depressive disorder.^105, 106^

## Modulation of the dopamine system and treatment of bipolar disorder

### Antidopaminergic drugs in the treatment of mania

Dopamine antagonists and partial agonists are increasingly used in the treatment of acute mania, bipolar depression and also as maintenance treatment.^107, 108, 109, 110^ Olanzapine, risperidone, quetiapine, aripiprazole, asenapine, ziprasidone and cariprazine have been approved by the Food and Drug Administration (FDA) as monotherapy for the treatment of acute mania.^111^ Pertinently, a network meta-analysis of 68 randomised controlled trials (RCT), involving 16 073 participants, addressed the efficacy of different drugs in the treatment of acute mania. This showed that dopamine antagonists have larger effect sizes (Haloperidol (standardised mean difference (SMD) relative to placebo: −0.56 (95% CI −0.69 to −0.43)), risperidone (−0.50 (−0.63 to −0.38)), olanzapine (−0.43 (−0.54 to −0.32)) than mood stabilisers for the treatment of acute mania (lithium) (−0.37 (−0.63 to −0.11)), carbamazepine (–0.36 (–0.60 to −0.11)), valproate (–0.20 (–0.37 to −0.04)).^14^

As the blockade of the dopamine D2/3 receptors is their common mechanism of action,^24^ it is likely that reduced dopamine neurotransmission at least contributes to the clinical efficacy of these drugs. This assumption is supported by the greater efficacy of the cis- but not the trans-isomer of clopenthixol in the treatment of manic symptoms,^112^ as the cis-isomer is a high affinity D2/3 receptor blocker while the trans-isomer has much lower affinity for D2/3 receptors.^44, 112^

### Use of antidopaminergic in the maintenance phase

The FDA have approved olanzapine as monotherapy, quetiapine and ziprasidone as adjunctive therapy, and aripiprazole and risperidone long acting injections as both adjunctive and monotherapy for the maintenance treatment of BD.^111^ Although lithium and valproate should be preferred, recent guidelines highlight that dopaminergic drugs have a place in maintenance treatment.^113^

### Action of non-D2/3 blocker mood stabilisers on dopamine pathways

Interestingly, it is possible that sodium valproate might exhibit antimanic effect through an action on the dopaminergic system.^44, 45^ In a study consisting of 13 manic patients, 2 weeks of sodium valproate was shown to decrease presynaptic dopamine synthesis capacity,^45^ although this was not correlated with clinical improvement. Interestingly D2/3 density remained essentially unchanged after valproate treatment,^44^ despite the reduction in dopamine synthesis capacity with valproate, which is anticipated to alter D2/3 availability. Taken together these two could either suggest valproate blocks the capacity of the D2 receptor to respond to reduced dopamine synthesis, or that the capacity of D2 receptors to respond is intrinsically impaired in bipolar disorder. Although both possibilities are speculative at this stage, the latter is particularly interesting as it suggests that a failure of D2 adaptation could contribute to the pathophysiology of bipolar disorder, making the dopamine system more vulnerable to dopamine transporter driven changes in presynaptic function.

Although the exact mechanism by which lithium acts remains unclear, it is known to modulate signalling pathways downstream of dopamine receptors. Preclinical studies have shown that lithium reverses dopamine dependent behaviour by acting through the protein kinase B (AKT)/glycogen synthase kinase 3 signalling cascade.^114^ Furthermore, lithium acts on the adenyl cyclase and phospho-inositide, as well as protein kinase C pathways, which are part of the intracellular signalling pathway downstream of dopamine receptors.^115^ In addition, micro-dialysis studies show a reduction in extracellular dopamine levels in lithium treated animals.^116, 117, 118^ In summary, there is evidence to suggest that some non-D2/3 blocking mood stabilisers also act to reduce dopamine transmission, either through reducing presynaptic dopamine synthesis capacity in the case of sodium valproate, or post-synaptic dopaminergic signal transduction in the case of lithium. The degree to which these effects explain the therapeutic efficacy of these compounds remains to be determined.

### Use of dopaminergic treatments for bipolar depression

Three double-blind placebo-controlled studies have shown that short-term use of pramipexole, a D2/D3 receptor agonist, is efficacious as an augmentation strategy for the treatment of bipolar depression and positive results have also been seen in other open-label studies.^119, 120, 121, 122, 123, 124, 125, 126, 127^ Although there have not been any RCTs investigating the efficacy of methylphenidate or amphetamines in bipolar depression, the available open-label and naturalistic studies point towards a benefit of stimulants in a selected group of patients with drowsiness and fatigue.^128, 129, 130, 131^ Randomised controlled studies and open-label reports with other stimulant like agents such as modafinil and its R-enantiomer, armodafinil also indicate efficacy in bipolar depression although the development programme for armodafinil failed.^132, 133, 134, 135, 136, 137, 138^ There is evidence to support the potential use of monoamine oxidase inhibitors such as tranylcypromine in bipolar depression.^139, 140^ It is speculated that tranylcypromine acts as dopamine releaser with about 1/10th the potency of amphetamine.^139, 140^ There are thus several lines of evidence suggesting that dopaminergic augmentation is beneficial in bipolar depression.

However, there is also evidence that dopamine antagonists are effective in the treatment of bipolar depression.^15^ The FDA has approved the combination of olanzapine and fluoxetine, as well as monotherapy with quetiapine or lurasidone, for the treatment of acute bipolar depression. A recent meta-analysis of 24 placebo-controlled trails (*n*=7307) revealed the following order of efficacy of drugs for the treatment of bipolar depression: olanzapine+fluoxetine⩾valproate>quetiapine>lurasidone>olanzapine, aripiprazole and carbamazepine.^141^ The analysis is interesting in showing that antidopaminergics on their own have efficacy in bipolar depression.

The evidence thus suggests that both dopaminergic agonists and dopaminergic blockers, are effective in treating bipolar depression. This presents a paradox for understanding the role of dopamine in bipolar depression. However, actions at receptors other than dopamine ones may underlie the efficacy of the dopamine blockers. For example, as well as all being D2/3 receptor blockers, olanzapine and quetiapine are both relatively high affinity 5HT_2A_ antagonists,^142^ whilst both lurasidone and aripiprazole have high affinity for 5HT_1A_ receptors.^142, 143^ Potentially supporting this explanation, there are no clinical trials reporting efficacy of pure D2/3 blockers in bipolar depression. Nevertheless, the doses used in the clinical trials correspond to the dose ranges associated with substantial dopamine D2/3 receptor occupancy,^24^ so dopamine receptor blockade is certainly compatible with antidepressant efficacy. The final consideration is that there has been a relatively small number of good quality RCTs for antidopaminergic treatment in bipolar depression, certainly in comparison to studies in mania. Studies that can directly test the mechanism underlying the mode of action of these drugs and pramipexole would be of great interest.

## Discussion

Our main findings for bipolar mania are that (i) there is consistent pharmacological evidence, especially from treatment studies, to support the hypothesis that a state of hyperdopaminergia can lead to mania; (ii) imaging studies support this hypothesis, with several studies reporting elevations in D2/3 receptor availability in psychotic mania and fMRI imaging evidence that identifies hyperactivity of the reward circuit in mania. Dopamine synthesis and receptor density appear to remain unchanged, at least in non-psychotic mania patients compared with HC.

**Figure 1:**
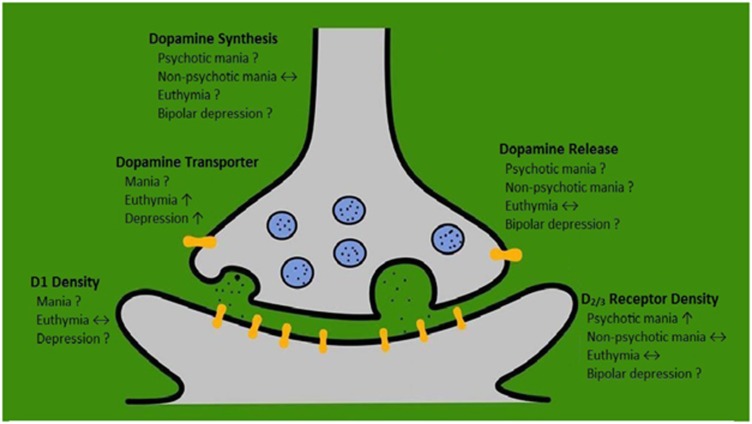
Summary of dopamine molecular imaging findings in bipolar disorder.

For bipolar depression (i) pharmacological evidence shows that dopamine agonists are potentially beneficial for bipolar depression, but the same seems true for dopamine blocking drugs; (ii) the imaging studies show replicated increases in dopamine transporter levels, but there is inconsistency and it is not clear if there are other alterations as well. Figure 1 summarises our main imaging findings for mania and depression.

Finally, post-mortem evidence suggests an upregulation of the D2/3 receptors in bipolar patients, but interpretation is limited by lack of information on phase of illness and medication status at the time of death.

### Implications for the dopamine hypothesis of bipolar disorder

More than four decades on from the early conceptualisations of the dopamine hypothesis of BD, it has stood the test of time, and the evidence for elements have strengthened. This is particularly the case for mania where the strength of the clinical trial evidence for the benefit of dopamine antagonists and partial agonists, supported by meta-analysis, would require an improbably large number of negative studies for reversal. Added to this there is now molecular and fMRI imaging evidence in bipolar disorder. Further, preclinical studies using optogenetic methods and knockout mice have shown that dopaminergic neuron activation leads to manic-like behaviour and it is linked to circadian gene expression.^31, 144^ Tentatively, these studies suggest elevated D2/3 receptor availability and a hyper-responsive reward system in ventral striatum in mania, and an increase in striatal dopamine transporter availability in bipolar depression. Dopamine neurotransmission in the striatum is primarily terminated by reuptake of dopamine into the presynaptic dopamine nerve terminal by dopamine transporters. Thus, an elevation in striatal D2/3 receptor availability in mania would lead to increased dopaminergic neurotransmission whilst increased striatal dopamine transporter levels in depression would lead to reduced dopaminergic function. This suggests a model in which elevated D2/3 receptor levels lead to altered reward processing and the development of mania, which is followed by a compensatory increase in dopamine transporter levels to reduce dopaminergic neurotransmission. However, if, over time, D2/3 receptor levels reduce but dopamine transporter levels do not normalise, this would then lead to reduced dopaminergic transmission, leading to depression and, in turn, a compensatory upregulation of D2/3 receptor levels, precipitating a further phase switch. One can see how a failure of homoeostatic regulation of the dopaminergic system could lead to cyclical periods of elevated and blunted dopaminergic neurotransmission. This model might apply most precisely to rapid cycling bipolar disorder. However it could be a component of all manic episodes, with other systems acting to disrupt the regular cyclicity that is so obvious in a substantial number of patients.

We recognise that this model is conjectural at this stage. Nevertheless, it makes predictions that can be tested empirically. In particular it predicts longitudinal changes in dopamine transporter and receptor levels linked to phase switches. Moreover it predicts that these changes and alterations in reward processing will pre-date symptom changes. However, many aspects of dopamine function in bipolar disorder are incompletely characterised. In particular baseline dopamine levels have not been measured in mania or depression. In addition, psychotic symptoms may also be present in manic patients, and psychosis *per se* may drive dopaminergic changes.^40, 145^ It is worth noting that, none of the studies have investigated dopamine function in mixed states. As approximately two thirds of depressed patients have concomitant manic symptoms,^146^ any theory needs to explain mixed states as well as mania and depression. It would be hard to account for mixed states by dysfunction in dopaminergic function alone. Interestingly, asenapine seems to be more effective than olanzapine for mixed episodes.^147^ As asenapine has higher affinity for 5HT2A than D2/3 receptors, which could suggest a role of serotonergic system in mixed episode states. Finally elevations in D_2/3_ receptor availability would be predicted to increase dopaminergic neurotransmission via the indirect pathway, and reduce response to cues,^148^ while the fMRI findings in mania do not seem to fit with this. However, the majority of the patients in these studies were treated with dopamine antagonists and mood stabilisers (Supplementary Table 2), which confounds simple interpretation of effects based on dopamine neurotransmission.

### Implications for treatment

If dopamine transmission is increased in mania, the use of D2/3 receptor blockers is logical, but alternative approaches, such as reducing dopamine synthesis and/or release, are alternative approaches that may be more effective and/or better tolerated if they could be sufficiently selective. Similarly, selective DAT blockade could be beneficial for BD depression, at the risk of precipitating mania. Finally mood stabilisation should improve homoeostatic regulation of dopaminergic neurotransmission, and this requires further evaluation and understanding.

### Limitations and future directions

The key limitation of the dopamine hypothesis remains that its strongest supporting evidence comes from pharmacological studies, which offer an indirect and sometimes imprecise approach to studying dopaminergic function. Reserpine, alpha-methyl-para-tyrosine, amphetamine and l-DOPA, for example, can affect neurotransmission of other monoamines, in particular norepinephrine, which could contribute to the clinical effects observed. By the same token, the efficacy of antidopaminergic drugs in mania may be due to actions at other neurotransmitter systems, although relatively selective dopamine blockers do appear to be effective. There is a relative paucity of evidence from more direct measures, such as molecular imaging studies, and thus conclusions remain tentative at this stage. Earlier molecular imaging studies on D2/3 density in mania were conducted using non-selective ligand [^11^C]N-methylspiperone, which has affinity to both D2/3 and 5HT2A receptors.^149^ Interpretation is further complicated by some studies not being restricted to one illness phase. Moreover, none of the studies addressed dopamine transporter availability in mania, dopamine release in mania or dopamine release in bipolar depression (summarised in Figure 1).

Although our model suggests euthymia is a state of normalised dopamine, based on a study demonstrating no significant alteration in the dopamine release paradigm in euthymic patients compared to controls,^50^ this is not consistent with the finding of elevated DAT in a euthymic state.^47^ It also remains unclear whether the dopamine hypothesis can completely explain bipolar depression and how dopaminergic dysregulation will be linked to the involvement of other neurotransmitter systems.

A critical limitation is the lack of longitudinal studies that investigate changes across phases of illness, including mixed states. Ideally future studies should focus on elucidating phase-related dopamine dysfunction by studying patients longitudinally in euthymic, manic and depressive episodes to determine the direction of causality. Clearly this will be difficult, although a focus on rapid cycling patients might make it feasible. Cross-sectional studies are more feasible and could test key elements of the dopamine hypothesis if well designed. Another key issue is why some antidopaminergics are effective for bipolar depression. Molecular imaging studies are needed to determine whether dopaminergic, serotonergic or other systems are involved in their mode of action. Finally, although studies have found an effect of valproate and lithium on dopaminergic function,^44, 115^ it is not established if this is their mechanism of action. Determining whether dopaminergic mechanisms are common across classes of medication would be an important advance in understanding. Finally, it remains unclear if hyperdopaminergic activity is specific to mania or psychosis because many of the manic patients in the studies had psychotic symptoms as well. It is interesting to note that in schizophrenia molecular imaging studies indicate there is an elevation in dopamine synthesis and release capacity, but unaltered dopamine transporter and D2/3 receptor availability in striatum.^40^ PET studies have also demonstrated elevated striatal dopamine turnover, but blunted cortical and midbrain dopamine release in schizophrenia.^150^ In addition, blunting of the fMRI signal during reward tasks is observed in schizophrenia.^85^ In contrast, studies in bipolar disorder suggest dopamine synthesis capacity is unaltered, at least in non-psychotic mania, but there is elevated D2/3 receptor availability in psychotic mania. However, direct comparisons of dopaminergic function in psychotic mania and schizophrenia as well as longitudinal studies of dopaminergic activity across various phases of illness are needed to determine if there are differences between mania and schizophrenia, and between mania and bipolar depression (Box 1).

## Conclusions

The dopamine hypothesis of bipolar disorder proposes that faulty homoeostasis between dopamine transporter and receptors underlies depressive and manic phases of the illness. The available evidence suggests elevated D2/3 receptor availability and a hyper-responsive reward system in mania, and possibly increased dopamine transporter availability in bipolar depression. Future longitudinal studies are needed to elucidate the precise phase-related changes in dopaminergic function and the specificity of alterations to mania over psychosis.

## Figures and Tables

**Table 1:** Post-mortem studies assessing the role of dopamine in bipolar disorder

*Measure of*	*Author*	*Bipolar patients/controls (*n*)*	*Bipolar patients/ controls age mean (s.d.) in years*	*Method*	*Area*	*Cause of death patients/ controls*	*Patients' medication*	*Post-mortem interval (h): patients/ controls*	*Results (BD relative to controls)*
Dopamine transporter	Rao *et al.*^134^	10/10	55 (s.e.m.: 6.6)/49 (s.e.m.: 4.3)	Western blot analysis, RNA isolation, RT-PCR	Prefrontal Cortex	Not mentioned	Lithium: 4 Valproate: 5 Rispiridone: 1 Carbamazepine: 1	21/20	↓ DAT protein and mRNA levels
	Lee *et al.*^135^	15/15	42.3 (9.3)/48 (10.7)	Coimmunoprecipitation and Western blot analyses	Striatum	Suicide-9/0 Non suicidal-6/15	Dopamine antagonist: 7	33/24	↔ DAT protein levels
Dopamine D1 receptor	Pantazopoulos *et al.*^37^	15/15	58.3/61.3	*In situ* hybridisation	Hippocampus	Suicide-3/0 Non suicidal- 12/15	Dopamine antagonist: 8	21/20	↑D1 mRNA expression in hippocampal sector CA2
	Kaalund *et al.*^34^	DLPFC 61/ 244 Hippocampus 31/192 Caudate nucleus 44/78	45/40 46/40 43/41	RNA extraction and Quantitative real-time PCR	DLPFC, hippocampus and caudate	Not mentioned	Dopamine antagonist: 17	Not mentioned	↑DRD1 in DLPFC and hippocampus. ↔ caudate nucleus
Dopamine D2 receptor	Zhan *et al.*^35^	32/34	45 (10)/43 (7)	Quantitative real-time PCR	Prefrontal cortex	Sucide-15/0 Non suicidal causes-17/34	Mean lifetime antidopaminergic exposure: 10 071 mg fluphenazine equivalent	37/29	↑ D2 mRNA level in prefrontal cortex
	Glantz *et al.*^36^	14/14	42.3 (11.7)/48.1 (10.7)	Western blot analysis and immunohistochemistry	Temporal Cortex	Suicide-9/0 Non suicidal-6/15	Lithium: 4 Dopamine antagonist: 8 Antidepressant: 8	33/24	↔
	Kaalund *et al.*^34^	DLPFC 61/ 244 Hippocampus 31/192 Caudate nucleus 44/78	45/40 46/40 43/41	RNA extraction and Quantitative real-time PCR	DLPFC, hippocampus and caudate	Not mentioned	Dopamine antagonist: 17	Not mentioned	↑D2L in DLPFC and hippocampus ↔ Caudate nucleus
	Lee *et al.*^135^	15/15	42.3 (9.3)/48 (10.7)	Coimmunoprecipitation and Western blot analyses	Striatum	Suicide-9/0 Non suicidal-6/15	Dopamine antagonist: 7	33/24	↔ D2 protein levels
D5 receptor	Knable *et al.*^38^	48 data sets		*In situ* hybridisation	Hippocampus	Not mentioned	Not mentioned	Not mentioned	↑ Dopamine D5 receptor RNA in dentate gyrus, CA1 and subiculum

Abbreviations: DAT, dopamine transporter; DLPFC, dorsolateral prefrontal cortex; PCR, polymerase chain reaction; RT-PCR, reverse transcription polymerase chain reaction.

**Table 2:** *In vivo* imaging studies assessing dopamine in mania

*Dopamine system studied*	*Author*	*Patients/controls (*n*)*	*Phase of illness-number of participants in the given phase*	*Medication*	Tracer*	*Primary outcome* *In patients compared with controls*	*Secondary outcome*
Dopamine synthesis	Yatham *et al.*^45^	13/14	Mania (Non-psychotic)-13	Sodium valproate	[^18^F]DOPA	↔ FDOPA uptake rate constants in the striatum. After 2–6 weeks treatment with sodium valporate, FDOPA rate constants - ↓	No correlations were found between YMRS and pre/post Ki values
D2/3 receptor availability	Yatham *et al.*^44^	13/14	Mania (Non-psychotic)-13	Sodium valproate	[^11^C]raclopride	↔ Striatal D2 density. After 2–6 weeks of treatment with sodium valproate no change in striatal D2 density (10 patients had second scan).	No correlation was found between D2 density and score of young mania rating scale. Similarly changes in the D2 density did not correlate with score in the YMRS
	Pearlson *et al.*^41^	14/12	Mania-11 (6 psychotic mania) Depression-3	All received two scans. Second scan was preceded by haloperidol lactate	[^11^C]N-methylspiperone	↑ D2 density in caudate and putamen	D2 availability directly correlated with psychotic symptom severity score, but no correlation with mania symptom rating
	Wong *et al.*^42^	14/24	Mania-11 (7 psychotic) Depression-3	Drug naive	N-[^11^C] methylspiperone ([^11^C]NMSP)	↑ D2 dopamine receptor density in caudate were seen in psychotic patients compared with non-psychotic patients and healthy controls	

Abbreviations: YMRS, Young's Mania Rating Scale; ↔, no significant difference.

*Further details of scan and patient characteristics are given in the Supplementary Material.

**Table 3:** *In vivo* imaging studies assessing dopamine in the depressive and euthymic phase of bipolar disease

*Dopamine system studied*	*Author*	*Patients/controls (*n*)*	*Phase of illness-number of participants in the given phase*	*Medication*	*Tracer*	*Primary outcome* *In patients compared with controls*	*Secondary outcome*
Vesicular monoamine transporter protein (VMAT-2)	Zubieta *et al.*^48^	16/16	Euthymic-16 (history of psychotic mania)	Valproic acid; lithium; carbamazepine; lamotrigine	[11C]dihydrotetrabenazine (DTBZ)	↔ Caudate ↑ Brainstem and thalamus	VMAT concentration in brainstem and thalamus positively correlated with the measure of frontal executive function
Dopamine release	Anand *et al.*^50^	13/13	Euthymic-13	7 Drug naive and 6 were on mood stabiliser: lithium (4) valproate (2). After first scan patients were administered amphetamine	[^123^I]IBZM	↔ In amphetamine-induced decrease in striatal [123I]IBZM binding.	In patients, amphetamine-induced decrease in [123I] IBZM binding did not correlate with post amphetamine YMRS score. In healthy controls, there was trend level correlation.
Dopamine transporter	Anand *et al.*^46^	11/13	Depressed-6 Euthymic-5	Drug naive	[^11^C]CFT	↓ DAT availability in caudate nucleus in patients	No correlations were found between YMRS/HDRS and D2 binding
	Amsterdam and Newberg^136^	5/46	Depression-5	Drug naive for at least a week	[^99^mTc] TRODAT-1	↑ Binding potential in the posterior putamen and in the left caudate region	
	Chang *et al.*^47^	17/17	Euthymic-17	Drug naive	[^99^mTc] TRODAT-1	↑ Striatal DAT availability	No significant difference in DAT availability between bipolar I and II.
D2/3 density	Anand *et al.*^50^	13/13	Euthymic-13	7 Drug naive and 6 were on mood stabiliser: lithium (4) valproate (2). After first scan patients were administered amphetamine	[^123^I]IBZM	↔ In striatal D2 receptor binding at baseline. ↔ In amphetamine-induced decrease in striatal [123I] IBZM binding.	In patients, amphetamine-induced decrease in [123I] IBZM binding did not correlate with post amphetamine YMRS score. In healthy controls, there was trend level correlation
D1 density	Suhara *et al.*^49^	10/21	Depressed-3 Manic-1 Euthymic-6	All except one were drug naive for at least one week before scan	[^11^C]-SCH23390	↓ D1, in the frontal cortex↔striatum	

Abbreviations: DAT, dopamine transporter; DVR, distribution volume ratio; HDRS, Hamilton Depression Rating Scale; YMRS, Young's Mania Rating Scale.

**Table 4:** Functional magnetic resonance imaging studies investigating bipolar patients vs healthy controls during monetary reward tasks

*Phase of illness*	*Author*	*BD* n *(%M)/ HC* n *(%M)*^a^	*fMRI task*	*VS ‘reward' activity in BD vs HC*		*Association with symptoms/dopamine antagonist medication*
				*Anticipation*	*Feedback*^a^	
Manic	Abler *et al.*^70^	12 BD I (58.3)/12 HC (58.3)	Monetary incentive task	↔ VS	↓ Left VS	
	Bermpohl *et al.*^71^	15 BD I (53.3)/26 HC (57.7)	MID	↔ VS	↔ VS	No association with antidopaminergic medication. Remitted BD (*n*=7, YMRS<8) OFC activation was similar to HC.
Euthymic	Yip *et al.*^82^	20 BD II /NOS (60)/20 HC (50)	MID	↔ VS (↓ r.DS)	↔ VS	No correlation between subsyndromal depressive symptoms (HDRS) and reward-related BOLD signal.
	Caseras *et al.*^72^	17 BD I (36)/15 BD II (40)/20 HC (35)	Card guessing task	BD I: ↔VS BD II: ↑VS	BD I:↑ left VS (trend) BD II:↔VS	During reward anticipation BD I had ↓VS activation vs BD II. At reward feedback BD I had ↑ activity in right VS vs BD II (not significant when excluding patients taking antidopaminergic medication or co-varying for medication load). VS activity not correlated with YMRS or HDRS.
	Mason *et al.*^76^	20 BD (18=BD I, 2=BD II) (50)/20 HC (45)	Roulette task	↑left VS (trend)	↑VS	Preferential activity for high probability rewards negatively correlated with impulsivity (DLPFC) and risk taking (DLPDC and VS)
	Trost *et al.*^81^	16 BD I (37.5)/16 HC (43.8)	Desire-reason dilemma	NA	↓VS	Task-appropriate bilateral VS suppression in BD correlated with antidopaminergic dose
	Nusslock *et al.*^77^	21 BD I (42.9)/20 HC (40)	Card guessing task	↑Right VS	↔VS	No association with antidopaminergic medication
	Dutra *et al.*^74^	24 BD I (37.5)/25 HC (40)	MID (no ‘loss' condition)	↔VS	↑VS	No association with antidopaminergic medication or symptom scores
	Linke *et al.*^75^	19 BD I (42.1)/19 HC (42.1)	Reversal learning	NA	↔VS	Negative correlation between medication load and mean activation of the right amygdala in response to reward in BD
Bipolar depression	Chase *et al.*^73^	23 BD I (17.4)/37 HC (32.4)	Card guessing task	↔VS	↔VS	AP associated with↓‘prediction error' VS signal. Illness duration associated with ↓ACC reward anticipation activation
	Satterthwaite *et al.*^79^	23 BD (21 BD I, 2 BD II) (37)/32 HC (51)	Monetary reward task	NA	↔VS	BDI correlated with diminished reward-related (win>loss) activation of bilateral VS, anterior and posterior cingulate, and anterior insula No effect of antidopaminergic dose on reward-related BOLD signal
	Redlich *et al.*^78^	33 BD I (51.5)/34 HC (52.9)	Card guessing task	Not analysed	↓VS	No association with medication load or symptom scores
Heterogeneous	Singh *et al.*^80^	24 adolescent BD1 (54)/24 HC (37)	MID	↔VS	↔VS	YMRS score associated with ↓VS activation during reward anticipation, when MID was preceded by ‘positive mood induction'

Abbreviations: ACC, anterior cingulate cortex; BD(I/II), bipolar disorder (I/II); BDI, beck depression inventory; DLPFC, dorsolateral prefrontal cortex; DS, dorsal striatum; fMRI, functional magnetic resonance imaging; HC, healthy controls; HDRS, Hamilton Depression Rating Scale; MDD, major depressive disorder; MID, monetary incentive delay task; NOS, not otherwise specified; YMRS, Young Mania Rating Scale.

Results presented are restricted to ventral striatum (VS), which has been most consistently implicated in event-related fMRI reward tasks. Values given as mean (s.d.) unless stated otherwise.

a Subject characteristics and reward feedback analysis contrasts are given in Supplementary Table 2.

**Table 5:** Summary of pharmacological evidence on dopamine dysfunction in the bipolar disorder

*Phase of illness*	*Drug*	*Mechanism of action*	*Effect*
Mania	Levodopa	Dopamine precursor	Induced mania and hypomania in parkinsonian patients
	Bromocriptine	Dopamine agonist	Induced mania in some patients who received it for postpartum galactorrhoea
	Amphetamine	Increases dopamine release	Induced mania-like symptom in healthy volunteers
	Dietary tyrosine depletion	Reduces dopamine level	Reduced manic symptoms in patients
	AMPT administration	Dopamine depletion	Reduced manic symptoms in patients and animal model
	Antidopaminergics	D2 blockers	Reduces manic symptoms
Euthymia	Olanzapine, quetiapine and ziprasidone	D2 blockade and 5HT agonism	Prolongs remission
	Mood stabilisers	Act on dopamine downstream pathways	Prolongs remission
Depression	Pramipexole	D2/3 agonist	Short-term efficacy in bipolar depression
	Methylphenidate and amphetamine	Increase dopamine release	Beneficial in group of bipolar depression patients with drowsiness and fatigue
	Olanzapine-fluoxetine combination, quetiapine and lurasidone	D2 blockade and serotonergic effects	Efficacy in bipolar depression

Abbreviation: AMPT, alpha-methyl-p-tyrosine.

## Future research direction

1. Animal models exhibiting bipolar depression-like phenotype need to be developed.
2. Development of better animal models of mood polarity switching coupled with longitudinal, translational assays (such as PET, fMRI and behavioural methods).
3. Longitudinal molecular imaging studies investigating dopaminergic function, particularly dopamine transporter and D2/3 receptor homoeostasis, in patients across different illness phases.
4. Trans-diagnostic comparisons of dopaminergic function in psychotic and non-psychotic mania and schizophrenia.
5. Imaging studies in mixed states and rapid cycling bipolar disorder.
6. Longitudinal studies investigating the effects of antidopaminergic and conventional mood stabilisers on dopaminergic pathways and the relationship between dopaminergic blockade and treatment response in bipolar disorder.
7. Future studies should also focus on integration of data from molecular imaging studies with functional MRI to elucidate specific anatomical circuits involved and the link to molecular changes.
8. Molecular imaging studies should investigate extra-striatal dopaminergic changes across different phases of bipolar disorder.Abbreviations: fMRI, functional magnetic resonance imaging; PET, positron emission tomography.
