# Supplementary material for: The dopamine hypothesis of bipolar affective disorder: the state of the art and implications for treatment
Source: Mol Psychiatry. 2017 Mar 14;22(5):666–79. doi: 10.1038/mp.2017.16 (PMC5401767; doi:10.1038/mp.2017.16)
Supplement: Supplementary Information [file mp201716x1.docx]

**Supplementary information:**

**Search strategy and selection criteria**

*The literature search was undertaken using PubMed with the following search terms: “dopamine”, “bipolar”, “mania”, “psychotic mania”, “euthymia”, “bipolar depression”, “positron emission tomography”, “PET”, “Single photon emission computed tomography”, “SPECT”, “post-mortem”, “antipsychotic”, “dopamine agonist”, “functional magnetic resonance imaging”, “fMRI”, “reward”. We identified 1441 studies from the search. We have reviewed all the post-mortem, positron emission tomography (PET) and functional magnetic resonance imaging (fMRI) studies we identified that reported original data in patients with bipolar disorder diagnosed using established criteria (eg using the Diagnostic and Statistical Manual) and a relevant control group. Seven post-mortem, nine molecular imaging and thirteen fMRI studies were identified. For the pharmacological studies and clinical trial data inclusion of all the studies was not possible due to space constraint. Instead, for these we have used systematic reviews and meta-analyses where available, and where not available have focused on randomised controlled studies.*

**Supplementary table 1: Subject and scan characteristics of PET studies**

| **Author & year** | **Patients/ Controls (Male: Patients/ controls)** | **Age Patients/ Controls Mean (SD) yrs** | **Duration of illness** | **ROI studied** | **Reference region** | **Tracer** | **Dose (total)** | **PET measure** |
| --- | --- | --- | --- | --- | --- | --- | --- | --- |
| Amsterdam & Newberg2007(Amsterdam and Newberg, 2007) | 5  /46 (2/22) | 40.9  /40 | Not mentioned | Striatum | Cerebellum | SPECT- [^99^mTc]  TRODAT-1 | 740 MBq | DVR |
| Anand et al 2000(Anand et al., 2000) | 13/13 (5/5) | 36 (7)/ 35 (8) | 14 years | Striatum | Cerebellum | SPECT- [^123^I]IBZM | 10 mCi | B_max_/K_d_ |
| Anand et al 2011(Anand et al., 2011) | 11/13 (6/ 5) | 27.3 (9.7)/ 27.5 (7.3) | 10 years | Striatum | Cerebellum | [^11^C]CFT | 740 MBq | B_max_/K_d_ |
| Chang et al 2010(Chang et al., 2010) | 17/17 (7/7) | 30.5 (8.1)/ 30.6 (8.3) | 6.9 years | Striatum | Occipital cortex | SPECT- [^99^mTc] TRODAT-1 | 740 MBq | DVR |
| Pearlson et al 1995(Pearlson et al., 1995) | 14/12 (9/9) | 40.2 (13.2)/ 28 (12.6) | 5.4 months (current episode) | Caudate | Cerebellum | [^11^C]N-methylspiperone | 7.4X1013 Bq/mmol | B_max_ |
| Suhara et al 1992(Suhara et al., 1992) | 10/21  (10/21) | Not mentioned/20-72 (range) | Not mentioned | Striatum,  Frontal cortex | Cerebellum | [^11^C]-SCH23390 | 385.7-679.69 MBq | k_3_/k_4_ |
| Wong et al 1997(Wong et al., 1997) | 14/24 (9/19) | Psychotic 41 (13)  Non psychotic  41 (14)/ 40 (22) | 5.4 months (current episode) | Caudate | Cerebellum | N-[^11^C] methylspiperone  ([^11^C]NMSP) | 1 µCi pmol^- I^ | B_max_ |
| Yatham et al 2002(Yatham et al., 2002b) | 13/14 (6/7) | 32.9 (12)/ 30.9 (11.4) | 4.04 weeks (current episode) | Striatum | Temporo-occipital cortex | [^18^F]DOPA | 185 MBq | K_i_ |
| Yatham et al 2002(Yatham et al., 2002a) | 13/14 (6/7) | 32.9 (12)/ 30.9 (11.4) | 4.04 weeks (current episode) | Striatum | Cerebellum | [11C]raclopride | 140 MBq | B_max_/K_d_ |
| Zubieta et al 2000 (Zubieta et al., 2000) | 16/16 (9/9) | 39 (13)/39(13) | 13.5 years | Caudate, brainstem and thalamus | Occipital cortex | [11C]Dihydrotetrabenazine | 666 MBq | DVR-1 |

**Supplementary table 2: Subject and study characteristics of fMRI studies**

| **Author & year** | **BD n (%M) / HC n (%M)** | **BD age / HC age** | **BD phase n** | **BD medication (n)** | **Mania / depression scores** | **ROI** | **Years of illness** | **Reward paradigm** | **Feedback contrast** |
| --- | --- | --- | --- | --- | --- | --- | --- | --- | --- |
| Abler et al., 2008 (Abler et al., 2008) | 12 BD1 (58.3) /12 HC (58.3) | 33.9 (11.2) /36.2 (11.2) | Manic 8, hypomanic 1, mixed 3, Psychotic 9. | AP: 12 (1 FGA) [CLPZ 375mg (397)]; Mood stab: 12; BZD: 5; AD: 0 | YMRS 21.8 (9.0) / MADRS 11.8 (15.4) | VS, VTA, brainstem | 12.8 (11.7) | Monetary incentive task with no monetary ‘loss’ condition | Reward> omission |
| Bermpohl et al., 2010(Bermpohl et al., 2010) | 15 BD1 (53.3) / 26 HC (57.7) | 38.6 (13.7) /38.7 (13.7) | Manic 15 | AP: 10 (1 FGA); Lithium: 8; Valproic acid: 5; Carbamazepine: 1; L-thyroxine: 3 | YMRS 18.9 (6.2) | VS, medial PFC, OFC | 16.2 (9.1) | MID | Reward> omission |
| Yip et al., 2015(Yip et al., 2015) | 20 BD2/NOS (60) / 20 HC (50) | 22.59 (4.0)/ 22.1 (2.6) | Euthymic | AP & MS naïve. | YMRS 1.2 (2.0) / HDRS 9.2 (6.9) | VS and DS | Not stated | MID | Reward > neutral condition |
| Caseras et al., 2013(Caseras et al., 2013) | 17 BD1 (36) / 15 BD2 (40) / 20 HC (35) | BD1: 42.8 (7.3) / BD2: 40.5 (8.1) / HC: 42.4 (6.0) | Euthymic | AP: (BD1=9. CLPZ= 428mg; BD2=3. CLPZ = 259mg ; MS: (BD1=12; BD2=10); AD: (BD1=6; BD2=6); Nil: (BD1=1; BD2=5) | YMRS BD1=3.2 (2.3) BD2=1.8 (2.8) / HDRS BD1=3.9 (3.9); BD2= 2.7 (2.9) | VS | BD1: 25.3  BD2: 21.6 | Card guessing task | Positive outcome > baseline |
| Mason et al., 2014(Mason et al., 2014) | 20 BD (18=BD1, 2=BD2) (50) / 20 HC (45) | 36.0 (8.3) / 33.3 (9.3) | Euthymic | AP: 0; Lithium: 8; Valproate: 5; Lamotrigine: 2; SSRI: 3; SSNRI: 3; BZD: 1; z-drug: 3; Nil: 4 | MAS12 3.2 (2.8) / HDRS 3.8 (3.0) | VS, VMPFC, DLPFC | Not stated | Roulette task | ↑>↓ reward magnitude; ↓ >↑ prob rewards. |
| Trost et al., 2014(Trost et al., 2014) | 16 BD1 (37.5) / 16 HC (43.8) | 35.6 (9.2) / 35.4 (9.9) | Euthymic 7, Mild dep. 8, mixed 2 | SGA: 8 (CLPZ 148.4 (191.7)); MS: 6; Lithium: 7; MS+Lithium: 1; AD 9; BZD: 0; Nil: 2. | YMRS 2.3 (5.6)/MADRS 10.0 (8.9) | VS, VTA, VLPFC | 11.1 (7.2) | Desire-reason dilemma | Reward cue in ‘desire context’ |
| Nusslock et al., 2012(Nusslock et al., 2012) | 21 BD1 (42.9) / 20 HC (40) | 31.5 (8.7) / 31.6 (6.9) | Euthymic | AP: 12; MS: 15; AD: 8; Dopaminergic AD: 3; BZD: 3 | YMRS 2.3 (2.5)/HDRS-25 6.4 (4.2) | OFC, VS | 13.4 (8.1) | Card guessing task | Reward> no-reward |
| Dutra et al., 2015(Dutra et al., 2015) | 24 BD1 (37.5) / 25 HC (40) | 31.4 (11.9) / 29.4 (8.8) | Euthymic | Medicated | YMRS 1.5 (1.7)/IDS-C 3.6 (2.1) | VS, OFC | 14.8 (11.5) | MID with no monetary ‘loss’ condition | Reward> omission |
| Linke et al., 2012(Linke et al., 2012) | 19 BD1 (42.1) / 19 HC (42.1) | 45 (10) / 45 (10) | Euthymic | Lithium: 4 ; Valproic acid: 2; Clomipramine: 1; Duloxetine 2; Sertraline & lithium 1; Venlafaxine & pregabalin: 1; opipramol & lithium 1; Nil: 7 | YMRS 0.9 (1.1) / HDRS 1.0 (1.5), BDI 2.9 (2.9) | Striatum, OFC, ACC, amygdala | 15.4 | Probabilistic reversal learning task | Reward> neutral trials |
| Chase et al., 2013(Chase et al., 2013) | 23 BD1 (17.4) / 37 HC (32.4) | 33.9 (8.5) / 33.1 (6.2) | Depressed | AP: 11; AD: 9; bupropion 2; MS: 13; anxiolytic: 5 | YMRS 4.0 (2.5)/HDRS-25 24.7 (8.0) | VS, left VLPFC, ACC | 17.3 (8.1) | Card guessing task | Parametric regressor: signed RPE |
| Satterthwaite et al., 2015(Satterthwaite et al., 2015) | 23 BD (21 BD1, 2 BD2) (37) / 32 HC (51) | 36.5 (11.9) / 39.5 (11.6) | Depressed | AP: 12 [CLPZ 342mg]; AD: 6; BZD: 7; Stimulants: 1; Lithium: 13; Lamotrigine: 8; Valproic Acid: 1. | BDI 21.8 (7.7) | VS, VMPFC, PCC, ACC, thalamus, insula | Not stated | Monetary reward task | Reward> loss outcome |
| Redlich et al., 2015(Redlich et al., 2015) | 33 BD1 (51.5) / 34 HC (52.9) | 38.1 (12.6) / 38.6, (12.3) | Depressed | AP: 24; MS: 17; SSNRI: 8; SSRI: 5; SNRI: 1; TCA: 2; MOAI: 2,; agomelatine: 1; Nil: 1 | YMRS 2.5 (2.4)/BDI 24.9 (8.6), HDRS 22.9 (4.6) | VS | 11.5 (10.3) | Card guessing task | Reward> control condition |
| Singh et al., 2013(Singh et al., 2013) | 24 adolescent BD1 (54) / 24 HC (37) | 15.7 (1.7)/ 15.0 (1.4) | Manic 6, mixed 5, depressed 9, euthymic 4 | SGA: 17; Lithium: 8; AD: 12; stimulants: 7 , Nil: 4. (Mean lifetime exposure 15.5 weeks) | YMRS 17.8(8.1)/CDRS 44.0 (14.8) | VS, ACC, amygdala, insula | 0.5 (0.3) | MID | Reward> omission |

Values given as mean (SD) unless stated otherwise. Abbreviations: ACC=anterior cingulate cortex, AD=antidepressant, AP=antidopaminergic, BD(I/II)=bipolar disorder (I/II), BDI= Beck depression inventory, BZD=benzodiazepine, CDRS=Childhood Depression Rating Scale, CLPZ=chlorpromazine-equivalent dose, DLPFC=dorsolateral prefrontal cortex, DS=dorsal striatum, FGA=first generation antidopaminergic, HC= healthy controls, HDRS(-25) = Hamilton Depression Rating Scale (25 item version), IDS-C=Inventory of Depressive Symptomatology, MDD=major depressive disorder, MADRS= Montgomery–Asberg Depression Rating Scale, MAOI = monoamine oxidase inhibitor, MID = monetary incentive delay task, MS=Mood stabilizer, NOS=not otherwise specified, OFC=orbitofrontal cortex, PCC=posterior cingulate cortex, PFC=prefrontal cortex, RPE=reward prediction error, SGA=second generation antidopaminergic, SNRI= Selective noradrenaline reuptake inhibitor, SSNRI=selective serotonin noradrenaline reuptake inhibitor, SSRI= Selective serotonin reuptake inhibitor, TCA=tricyclic antidepressant, VL/VM-PFC = ventrolateral/ventromedial prefrontal cortex, VS=ventral striatum, VTA=ventral tegmental area, YMRS=Young Mania Rating Scale

Abler, B., Greenhouse, I., Ongur, D., Walter, H., Heckers, S., 2008. Abnormal reward system activation in mania. Neuropsychopharmacology : official publication of the American College of Neuropsychopharmacology 33, 2217-2227.

Amsterdam, J.D., Newberg, A.B., 2007. A preliminary study of dopamine transporter binding in bipolar and unipolar depressed patients and healthy controls. Neuropsychobiology 55, 167-170.

Anand, A., Barkay, G., Dzemidzic, M., Albrecht, D., Karne, H., Zheng, Q.H., Hutchins, G.D., Normandin, M.D., Yoder, K.K., 2011. Striatal dopamine transporter availability in unmedicated bipolar disorder. Bipolar disorders 13, 406-413.

Anand, A., Verhoeff, P., Seneca, N., Zoghbi, S.S., Seibyl, J.P., Charney, D.S., Innis, R.B., 2000. Brain SPECT imaging of amphetamine-induced dopamine release in euthymic bipolar disorder patients. The American journal of psychiatry 157, 1108-1114.

Bermpohl, F., Kahnt, T., Dalanay, U., Hägele, C., Sajonz, B., Wegner, T., Stoy, M., Adli, M., Krüger, S., Wrase, J., Ströhle, A., Bauer, M., Heinz, A., 2010. Altered representation of expected value in the orbitofrontal cortex in Mania. Human Brain Mapping 31, 958-969.

Caseras, X., Lawrence, N.S., Murphy, K., Wise, R.G., Phillips, M.L., 2013. Ventral striatum activity in response to reward: Differences between bipolar i and II disorders. American Journal of Psychiatry 170, 533-541.

Chang, T.T., Yeh, T.L., Chiu, N.T., Chen, P.S., Huang, H.Y., Yang, Y.K., Lee, I.H., Lu, R.B., 2010. Higher striatal dopamine transporters in euthymic patients with bipolar disorder: a SPECT study with [Tc] TRODAT-1. Bipolar disorders 12, 102-106.

Chase, H.W., Nusslock, R., Almeida, J.R., Forbes, E.E., Labarbara, E.J., Phillips, M.L., 2013. Dissociable patterns of abnormal frontal cortical activation during anticipation of an uncertain reward or loss in bipolar versus major depression. Bipolar disorders 15, 839-854.

Dutra, S., Cunningham, W., Kober, H., Gruber, J., 2015. Elevated Striatal Reactivity Across Monetary and Social Rewards in Bipolar I Disorder. Journal of Abnormal Psychology 124, 890-904.

Linke, J., King, A.V., Rietschel, M., Strohmaier, J., Hennerici, M., Gass, A., Meyer-Lindenberg, A., Wessa, M., 2012. Increased medial orbitofrontal and amygdala activation: Eidence for a systems-level endophenotype of bipolar I disorder. The American journal of psychiatry 169, 316-325.

Mason, L., O'Sullivan, N., Montaldi, D., Bentall, R.P., El-Deredy, W., 2014. Decision-making and trait impulsivity in bipolar disorder are associated with reduced prefrontal regulation of striatal reward valuation. Brain : a journal of neurology 137, 2346-2355.

Nusslock, R., Almeida, J., Forbes, E., Versace, A., Frank, E., LeBarbara, E., Klein, C., Phillips, M.L., 2012. Waiting to win: elevated striatal and orbitofrontal cortical activity during reward anticipation in euthymic bipolar disorder adults. Bipolar.Disord. 14, 249-260.

Pearlson, G.D., Wong, D.F., Tune, L.E., Ross, C.A., Chase, G.A., Links, J.M., Dannals, R.F., Wilson, A.A., Ravert, H.T., Wagner, H.N., Jr., et al., 1995. In vivo D2 dopamine receptor density in psychotic and nonpsychotic patients with bipolar disorder. Archives of general psychiatry 52, 471-477.

Redlich, R., Dohm, K., Grotegerd, D., Opel, N., Zwitserlood, P., Heindel, W., Arolt, V., Kugel, H., Dannlowski, U., 2015. Reward Processing in Unipolar and Bipolar Depression: A Functional MRI Study. Neuropsychopharmacology : official publication of the American College of Neuropsychopharmacology 40, 1-28.

Satterthwaite, T.D., Kable, J.W., Vandekar, L., Katchmar, N., Bassett, D.S., Baldassano, C.F., Ruparel, K., Elliott, M.a., Sheline, Y.I., Gur, R.C.R.E., Gur, R.C.R.E., Davatzikos, C., Leibenluft, E., Thase, M.E., Wolf, D.H., 2015. Common and Dissociable Dysfunction of the Reward System in Bipolar and Unipolar Depression Reward Dysfunction in Depression. Neuropsychopharmacology : official publication of the American College of Neuropsychopharmacology 40, 1-11.

Singh, M.K., Chang, K.D., Kelley, R.G., Cui, X., Sherdell, L., Howe, M.E., Gotlib, I.H., Reiss, A.L., 2013. Reward Processing in Adolescents With Bipolar I Disorder. Journal of the American Academy of Child & Adolescent Psychiatry 52, 68-83.

Suhara, T., Nakayama, K., Inoue, O., Fukuda, H., Shimizu, M., Mori, A., Tateno, Y., 1992. D1 dopamine receptor binding in mood disorders measured by positron emission tomography. Psychopharmacology 106, 14-18.

Trost, S., Diekhof, E.K., Zvonik, K., Lewandowski, M., Usher, J., Keil, M., Zilles, D., Falkai, P., Dechent, P., Gruber, O., 2014. Disturbed Anterior Prefrontal Control of the Mesolimbic Reward System and Increased Impulsivity in Bipolar Disorder. Neuropsychopharmacology : official publication of the American College of Neuropsychopharmacology 39, 1914-1923.

Wong, D.F., Pearlson, G.D., Tune, L.E., Young, L.T., Meltzer, C.C., Dannals, R.F., Ravert, H.T., Reith, J., Kuhar, M.J., Gjedde, A., 1997. Quantification of neuroreceptors in the living human brain: IV. Effect of aging and elevations of D2-like receptors in schizophrenia and bipolar illness. Journal of cerebral blood flow and metabolism : official journal of the International Society of Cerebral Blood Flow and Metabolism 17, 331-342.

Yatham, L.N., Liddle, P.F., Lam, R.W., Shiah, I.S., Lane, C., Stoessl, A.J., Sossi, V., Ruth, T.J., 2002a. PET study of the effects of valproate on dopamine D(2) receptors in neuroleptic- and mood-stabilizer-naive patients with nonpsychotic mania. The American journal of psychiatry 159, 1718-1723.

Yatham, L.N., Liddle, P.F., Shiah, I.S., Lam, R.W., Ngan, E., Scarrow, G., Imperial, M., Stoessl, J., Sossi, V., Ruth, T.J., 2002b. PET study of [(18)F]6-fluoro-L-dopa uptake in neuroleptic- and mood-stabilizer-naive first-episode nonpsychotic mania: effects of treatment with divalproex sodium. The American journal of psychiatry 159, 768-774.

Yip, S.W., Worhunsky, P.D., Rogers, R.D., Goodwin, G.M., 2015. Hypoactivation of the Ventral and Dorsal Striatum During Reward and Loss Anticipation in Antipsychotic and Mood Stabilizer-Naive Bipolar Disorder. Neuropsychopharmacology : official publication of the American College of Neuropsychopharmacology 40, 658-666.

Zubieta, J.K., Huguelet, P., Ohl, L.E., Koeppe, R.A., Kilbourn, M.R., Carr, J.M., Giordani, B.J., Frey, K.A., 2000. High vesicular monoamine transporter binding in asymptomatic bipolar I disorder: sex differences and cognitive correlates. The American journal of psychiatry 157, 1619-1628.
